# Development and Validation of a Simple, Rapid and Inexpensive PCR-RFLP Method for Genotyping of Common IL28B Polymorphisms: A Useful Pharmacogenetic Tool for Prediction of Hepatitis C Treatment Response

**DOI:** 10.5812/hepatmon.849

**Published:** 2012-03-28

**Authors:** Heidar Sharafi, Ali Pouryasin, Seyed Moayed Alavian, Bita Behnava, Maryam Keshvari, Leila Mehrnoush, Shima Salimi, Osveh Kheradvar

**Affiliations:** 1 Armin Pathobiology Laboratory, Tehran, IR Iran; 2 Tehran Hepatitis Cohort (THC) Study, Tehran, IR Iran; 3 Department of Genetics, Islamic Azad University, Arsanjan branch, Arsanjan, IR Iran; 4 Baqiyatallah Research Center for Gastroenterology and Liver Diseases, Baqiyatallah University of Medical Sciences, Tehran, IR Iran; 5 Iranian Blood Transfusion Organisation, Tehran, IR Iran

**Keywords:** Polymorphism, Genetic, Hepatitis C, IL28B Protein, Human, Iran, Polymorphism, Restriction Fragment Length

## Abstract

**Background:**

In 2009, 3 genome-wide association studies implicated IL28B single-nucleotide polymorphisms (SNPs) as the strongest genetic pretreatment predictor of sustained virological response (SVR) in hepatitis C infection. Recently, the American Association for the Study of Liver Diseases (AASLD) and the European Association for the Study of the Liver (EASL) included IL28B testing in their guidelines.

**Objectives:**

The main aim of this study was to develop and validate a simple, rapid, and inexpensive polymerase chain reaction-restriction fragment length polymorphism (PCR-RFLP) method for genotyping of common IL28B polymorphisms (rs12979860 and rs8099917).

**Patients and Methods:**

Two methods were developed to genotype common IL28B polymorphisms: 1) PCR-sequencing as a reference method and 2) PCR-RFLP as a rapid and inexpensive method. Both polymorphisms were genotyped in 104 Iranian hepatitis C patients by both methods simultaneously. To validate the PCR-RFLP method, the PCR-RFLP genotyping results should be 100% concordant with the PCR-sequencing results.

**Results:**

Genotyping of rs12979860 and rs8099917 by PCR-RFLP was concordant with PCR-sequencing in 104 (100%) individuals. The analytical sensitivity and specificity of the PCR-RFLP method for genotyping of both SNPs are 100%. Among these 104 patients with chronic hepatitis C, the frequency of the rs12979860 CC, CT and TT genotypes were 40.4%, 47.1% and 12.5% and the frequency of the rs8099917 TT, GT and GG genotypes were 59.6%, 35.6% and 4.8%, respectively. Also, three IL28B haplotypes (rs12979860-rs8099917) were found among our patients including C-T, T-G and T-T with 63.9%, 22.6% and 13.5% frequency, respectively. C-G haplotype was absent in all of our patients.

**Conclusions:**

We have developed a validated, fast, and simple PCR-RFLP method for genotyping of common IL28B SNPs that is more cost-effective than sequencing.

## 1. Background

Chronic hepatitis C infection remains a global public health concern and has a prevalence of approximately 2.2% globally [1]. Chronic hepatitis C infection is a major cause of cirrhosis and hepatocellular carcinoma [2]. The standard of care (SOC) for treatment of chronic hepatitis C infection is pegylated interferon α plus ribavirin, which leads to sustained virological response (SVR) in about 40%-60% of patients with hepatitis C virus (HCV) genotype 1 [3]. It appears that Iranian HCV-infected patients respond better to SOC treatment [4]. The cost of SOC treatment is relatively high, and this type of treatment may cause many undesirable side effects [5]. Various factors have been proposed as predictors of SVR, including HCV genotype, HCV RNA levels, the dose and duration of therapy, body mass index, age, insulin resistance, gender, stage of fibrosis, and co-infection with other hepatitis viruses or HIV [6]. In 2009, 3 genome-wide association studies (GWASs) demonstrated an impact of single-nucleotide polymorphisms (SNPs) in the spanning region between IL28B and IL28A on interferon-based hepatitis C clearance [7][8][9], implicating rs12979860 and rs8099917 as the strongest genetic determinants of SVR. According to these studies, the rs12979860 C and rs8099917 T alleles are associated with a higher rate of SVR, whereas the rs12979860 T and rs8099917 G alleles are linked to a lower rate of SVR and a higher rate of treatment failure. Also, it has been shown that the rs12979860 CC and rs8099917 TT genotypes are strongly associated with spontaneous resolution of HCV infection [10][11]. There are various aspects of the clinical utilization of IL28B testing, but it appears that it can help clinicians in decision-making with regard to treatment [12][13]. Recently, the American Association for the Study of Liver Diseases (AASLD) included IL28B testing in its guidelines [14], with a recommendation grade of Class 2a and Level B. Several methods, such as DNA sequencing, Taqman® allelic discrimination assay, pyrosequencing, polymerase chain reaction-restriction fragment length polymorphism (PCR-RFLP), and allele-specific PCR, can be applied for genotyping of SNPs, such as IL28B polymorphisms [15]. These methods vary in features, such as cost-effectiveness, reliability, equipment needs, and technical issues.

PCR-RFLP, also known as cleaved amplified polymorphic sequences (CAPS), is a simple, reliable, relatively fast, and inexpensive method with minimum equipment requirements. RFLP is based on the creation or deletion of recognition site of a restriction endonuclease by nucleotide variations in the polymorphic site; thus, digestion of the PCR product containing the polymorphism with an appropriate restriction endonuclease results in disparate electrophoretic patterns by polymorphism genotype.

## 2. Objectives

The aim of this study was to develop and validate a simple, rapid, and inexpensive PCR-RFLP method for genotyping of common IL28B polymorphisms (rs12979860 and rs8099917). Also, based on the genotyping results, we report the genotype and related haplotype frequency of IL28B SNPs (rs12979860 and rs8099917) in Iranian patients with chronic hepatitis C.

## 3. Patients and Methods

### 3.1. Study Population

We selected 104 consecutive and unrelated Iranian chronic hepatitis C patients (88 males, 16 females) who were treated at Tehran Hepatitis Center (Tehran, Iran) and referred to Armin Pathobiology Laboratory (Tehran, Iran). All patients were positive for anti-HCV antibody, as confirmed by recombinant immunoblot assay (RIBA).

### 3.2. IL28B Genotyping by PCR-Sequencing

Genomic DNA was extracted from 200 µl buffy coat using the QIAamp® DNA Blood Mini Kit (Qiagen, Hilden, Germany) according to the manufacturer's instructions. Primers for PCR-sequencing and PCR-RFLP of rs12979860 and rs8099917 polymorphisms were designed using Primer-Blast [16], considering the homology in the region between IL28B and IL28A genes. The primers are listed in Table 1. PCR was performed using Accupower® PCR PreMix (Bioneer, South Korea) with a 20 µl reaction tube type. Briefly, 100-300 ng of genomic DNA was amplified with 10 pmol of rs12-F and rs12-R primers for the genomic region containing rs12979860 and 10 pmol of rs80-F and rs80-R primers for the genomic region containing rs8099917. The PCR temperature profile comprised 94°C for 5 min; 35 cycles of 94°C for 20 s, 66°C for 20 s, and 72°C for 20 s; and 72°C for 5 min. The resulting PCR products were bidirectionally sequenced using BigDye® Terminator V3.1 Cycle Sequencing Kits (Applied Biosystems, Foster City, CA, USA) in a 3130 Genetic Analyzer (Applied Biosystems, Foster City, CA, USA) according to the manufacturer's instructions. Sequencing results were analyzed using Chromas Lite Version 2.01 (Technelysium).

**Table 1:** Primers for PCR-Sequencing and PCR-RFLP Genotyping of rs12979860 and rs8099917 SNPs

**SNP**	**Primer Name**	**Sequence**
rs12979860		
	rs12-F	5’ GCGGAAGGAGCAGTTGCGCT 3’
	rs12-R	5’ GTGCCTTCACGCTCCGAGCA 3’
	bst-R	5’ GGGGCTTTGCTGGGGGAGTG 3’
rs8099917		
	rs80-F	5’ CCCACTTCTGGAACAAATCGTCCC 3’
	rs80-R	5’ TCTCCTCCCCAAGTCAGGCAACC 3’

### 3.3. IL28B Genotyping by PCR-RFLP

The PCR primers that were used for PCR-RFLP genotyping of rs8099917 SNP were the same as those for PCR-sequencing of this polymorphism, but for PCR-RFLP of rs12979860 SNP, the rs12-F primer was used as the forward primer and bst-R was the reverse primer. The DNA amount and primer concentration for the PCR mixture and the temperature profile for the PCR amplification were the same as those for PCR-sequencing method. For RFLP analysis, the PCR product of rs12979860 was digested with 10 units of Bsh1236I (BstUI) restriction endonuclease (Fermentas, Vilnius, Lithuania), and the PCR product of rs8099917 was digested with 10 units of BseMI (BsrDI) restriction endonuclease (Fermentas, Vilnius, Lithuania) for one hour. The digestion products were separated on a 3% agarose gel alongside the GeneRulerTM 100 bp DNA Ladder (Fermentas, Vilnius, Lithuania). In each PCR-RFLP genotyping run, as a control for the enzymatic activity of Bsh1236I and BseMI, respectively, rs12979860 CC DNA and rs8099917 GG DNA were included.

### 3.4. Validation Protocol

For validation of the PCR-RFLP method, the PCR-RFLP genotyping results of rs12979860 and rs8099917 should be 100% concordant with the DNA sequencing results. Also, the reproducibility of the PCR-RFLP method was assessed by repeating the genotyping of both SNPs for 50 patients in an independent experiment.

### 3.5. Hardy-Weinberg Equilibrium and Haplotype Estimation

The genotype distribution of rs12979860 and rs8099917 was tested for Hardy-Weinberg equilibrium. Also, haplotype estimation of IL28B was performed using Arlequin version 3.5 software [17] and the expectation-maximization algorithm.

## 4. Results

### 4.1. IL28B Genomic Region and PCR Primer Design

**Figure 1:**
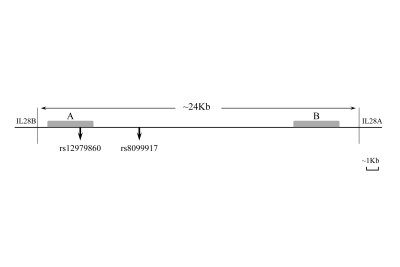
The Genomic Region Spanning Between IL28B and IL28A Genes, and the Location of Common IL28B Polymorphisms. IL28B rs12979860 SNP resides in the region A, which is homologous to region B

**Figure 2:**
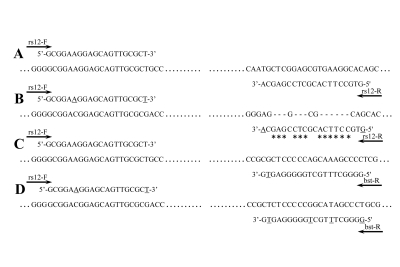
Primers for PCR Amplification of rs12979860 and Their Probable Binding Sites on IL28B Flanking Region. A) rs12979860 PCR-sequencing primers and the binding site on their specific genomic region; B) rs12979860 PCR-sequencing primers and the undesirable and probable primer binding site on the rs12979860 homologous genomic region; C) rs12979860 PCR-RFLP primers and the binding site on their specific genomic region; D) rs12979860 PCR-RFLP primers and the undesirable and probable primer binding site on the rs12979860 homologous genomic region; The underlined nucleotides represent the primer mismatches. The primer nucleotides denoted by asterisk are not present in the probable primer binding site

rs12979860 and rs8099917 are located in the 24 Kb genomic region between IL28B and IL28A genes. This genomic region contains few homologous sequences. rs12979860 is located 3 kb upstream of IL28B in one of these homologous regions, and rs8099917 is located 8 kb upstream of IL28B, but we did not find any homology in its genomic region (Figure 1). PCR primers were designed considering the homology in the rs12979860 genomic region. As we show in Figure 2B, the complementary sequence of rs12-R primer presentes just in about 30% sequence of the rs12979860 homologous region (the genomic region which is shown as B in the Figure 1); thus, the rs12-F and rs12-R primer pair binds specifically to the rs12979860 genomic region. Based on the specific primers which were designed for PCR-sequencing of rs12979860, we applied PCR-sequencing as the reference method. We designed another reverse primer (bst-R) for PCR-RFLP genotyping of rs12979860. As we show in Figure 2C, this primer binds to its specific site with a conventional mismatch, which results in better separation of digested PCR products by electrophoresis. Among the primers that were designed for PCR-RFLP genotyping of rs12979860, we selected a primer pair with the maximum number of mismatches to the homologous genomic region. Due to the absence of homology in the rs8099917 genomic region, the primer design for this SNP did not require any special consideration.

### 4.2. PCR-Sequencing Set-Up

**Figure 3:**
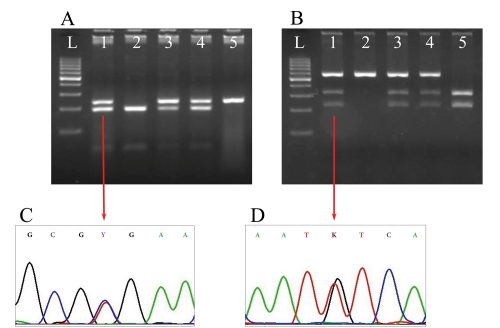
PCR-RFLP electrophoresis Results and PCR-Sequencing Chromatograms. A) rs12979860 PCR-RFLP genotyping, lanes no. 1, 3 and 4 were genotyped CT, lane no. 2 was genotyped CC and lane no. 5 was genotyped TT; B) rs8099917 PCR-RFLP Genotyping, lanes no. 1, 3 and 4 were genotyped GT, lane no. 2 was genotyped TT and lane no. 5 was genotyped GG; C) rs12979860 CT sequencing chromatogram; D) rs8099917 GT sequencing chromatogram. L, 100 bp DNA ladder

Amplification of genomic DNA with rs12-F and rs12-R primers produced a 746 bp DNA fragment. In this fragment, rs12979860 is located 197 bp from the 5' end of the forward primer (rs12-F). Figure 3C shows the rs12979860 CT sequencing chromatogram.

Amplification of genomic DNA with rs80-F and rs80-R primers yielded a 552 bp DNA fragment. In this fragment, rs8099917 is located 229 bp from the 5' end of the forward primer (rs80-F). Figure 3D shows the rs8099917 GT sequencing chromatogram.

### 4.3. PCR-RFLP Set-Up

We used the rs12-F and bst-R primer pair for PCR-RFLP genotyping of rs12979860, which amplified a 241 bp DNA fragment. Digestion of this product with Bsh1236I in CC individuals produced 2 fragments of 196 and 45 bp; 3 fragments of 241, 196, and 45 bp in CT individuals; and a 241 bp fragment in TT individuals (Figure 3A). For PCR-RFLP genotyping of rs8099917, we used the same PCR product as that used for PCR-sequencing of rs8099917. Digestion of this 552 bp PCR product with BseMI in TT individuals produced a 552 bp DNA fragment; 3 fragments of 552, 322, and 230 bp in GT individuals; and 2 fragments of 322 and 230 bp in GG individuals (Figure 3B).

### 4.4. Validation Results

Genotyping of rs12979860 and rs8099917 by PCR-RFLP was concordant with PCR-sequencing in all 104 (100%) individuals. The analytical sensitivity and specificity of the developed PCR-RFLP method for genotyping of rs12979860 and rs8099917 are 100%. PCR-RFLP genotyping of rs12979860 and rs8099917 was reproducible in all 50 (100%) patients in the duplication test. The genotype and allele frequencies of both SNPs are presented in Table 2. Also, 6 compound rs12979860/rs8099917 genotypes were observed among our patients-CC/TT, CT/GT, CT/TT, TT/GT, TT/GG, and TT/TT-at the following frequencies: 40.4%, 30.8%, 16.3%, 4.8%, 4.8%, and 2.9%, respectively.

**Table 2:** rs12979860 and rs8099917 Genotype and Allele Frequency

	**Frequency, %**
**rs12979860**	
Genotype	
CC	40.4
CT	47.1
TT	12.5
Allele	
C	63.9
T	36.1
**rs8099917**	
Genotype	
TT	59.6
GT	35.6
GG	4.8
Allele	
T	77.4
G	22.6

### 4.5. Hardy-Weinberg Equilibrium and Haplotype Estimation Results

Both rs12979860 and rs8099917 genotype distributions were in Hardy-Weinberg equilibrium (P = 0.82 and P = 0.86, respectively). According to the haplotype estimation, 3 rs12979860-rs8099917 haplotypes were noted in our patients_C-T, T-G, and T-T_at frequencies of 63.9%, 22.6%, and 13.5%, respectively. The C-G haplotype was absent from our patients.

## 5. Discussion

The identification of IL28B SNPs as the strongest baseline predictor of SVR by recent GWASs [7][8][9] has improved our knowledge on the challenging interindividual variations in interferon-based treatment outcomes in HCV infected patients. The disparate distributions of IL28B genotypes in Caucasians, Asians, and Africans explains the different rates of SVR in these populations. Today, IL28B genotyping is included in the AASLD and the European Association for the Study of the Liver (EASL) guidelines [6][14]. As a result, physicians should consider IL28B testing in hepatitis C patients, and laboratories should include IL28B genotyping in their hepatitis C test panel. The PCR-RFLP method that we designed can be used to genotype IL28B in less than 5 hours in nearly every laboratory, with minimum equipment requirements for molecular diagnosis. The PCR-RFLP method passed all of the criteria for validation and reached 100% analytical specificity and sensitivity. The PCR-RFLP method for IL28B genotyping is more cost-effective than DNA sequencing and does not need the expensive capital equipments that are required for DNA sequencing. In this study on Iranian patients with chronic hepatitis C, the rs12979860 C and rs8099917 T allele frequencies were 63.9% and 77.4%, respectively, similar to the 63.4% rs12979860 C allele frequency by Ge et al. in 871 European-American patients with chronic hepatitis C infection [7] and the 76.5% rs8099917 T allele frequency by Rauch et al. in 1015 patients (white population) with chronic hepatitis C infection [18]. Also, we described haplotypes that were tagged by rs12979860 and rs8099917 SNPs. The impact of these haplotypes on outcomes of interferon-based treatment will be reported by our study group soon. In conclusion, we have developed a validated, fast, and simple PCR-RFLP method for genotyping of IL28B rs12979860 and rs8099917 SNPs that is more cost-effective than sequencing.
